# Effect of Organic Cation on Optical Properties of [A]Mn(H_2_POO)_3_ Hybrid Perovskites

**DOI:** 10.3390/molecules27248953

**Published:** 2022-12-15

**Authors:** Dagmara Stefańska

**Affiliations:** Institute of Low Temperature and Structure Research, Polish Academy of Sciences, Okólna 2, 50-422 Wroclaw, Poland; d.stefanska@intibs.pl

**Keywords:** hybrid perovskites, hypophosphites, luminescence properties, manganese

## Abstract

Hybrid organic–inorganic compounds crystallizing in a three-dimensional (3D) perovskite-type architecture have attracted considerable attention due to their multifunctional properties. One of the most intriguing groups is perovskites with hypophosphite linkers. Herein, the optical properties of six hybrid hypophosphite perovskites containing manganese ions are presented. The band gaps of these compounds, as well as the luminescence properties of the octahedrally coordinated Mn^2+^ ions associated with the ^4^T_1g_(G) → ^6^A_1g_(S) transition are shown to be dependent on the organic cation type and Goldschmidt tolerance factor. Thus, a correlation between essential structural features of Mn-based hybrid hypophosphites and their optical properties was observed. Additionally, the broad infrared luminescence of the studied compounds was examined for potential application in an indoor lighting system for plant growth.

## 1. Introduction

Hybrid organic–inorganic compounds with a three-dimensional (3D) perovskite structure constitute a very large family of materials. Their general formula is ABX_3_, where A is an alkali metal or an organic cation, B is a divalent metal cation, and X denotes an organic or inorganic anion (halide, HCOO^−^, N_3_^−^, CN^−^, N(CN)_2_^−^, or H_2_PO_2_^−^). The 3D perovskite structure consists of corner-sharing BX_6_ (X = Cl, Br, I, O, or N) octahedral units. Metal ions and linkers form a three-dimensional framework, and organic cations are accommodated inside the created voids. Due to their unique physical phenomena, such as ferroelectricity, multiferroicity, and dielectricity, as well as magnetic and luminescent properties, these compounds have been extensively studied in recent years [1,2,3,4,5,6,7,8,9,10,11]. Hybrid organic–inorganic perovskites (HOIPs) are frequently called multifunctional materials because they exhibit several physical phenomena at the same time [12,13,14,15,16,17,18,19]. As a consequence, HOIPs offer a wide range of potential applications for catalysis, gas storage, drug delivery, nonlinear optics, solid-state lighting, luminescent sensors, temperature sensors, biomedical photonics, and numerous other utilizations [12,13,15,19,20,21,22].

Lead halides are one of the most commonly investigated families of HOIPs [4,12,23,24,25]. For instance, lead halides containing methylammonium (MA^+)^ or formamidinium (FA^+^) cations attract great interest due to their promising application in solar cells [26,27,28,29], whereas methylhydrazinium (MHy^+^) lead halides have been reported to exhibit multiple nonlinear optical phenomena [4,24,30]. It is worth noting that the band gap value (*E_g_*) and the position of the free exciton (FE) emission depend both on the halide used and the type of organic cation. Generally, the A-site organic cation is often ordered at low temperatures and becomes disordered as the temperature rises. Furthermore, organic cations have different sizes, shapes, and the ability to form hydrogen bonds (HBs) with the metal-ligand framework [31,32,33].

Three-dimensional hypophosphites are a relatively novel family of HOIPs. They were obtained for the first time in 2017 [34]. Unfortunately, the number of hypophosphite perovskites discovered is still low [34,35,36,37,38]. To date, only a few manganese, cadmium, and magnesium hypophosphite analogs have been reported [34,35,36,37,38]. Structural data revealed that metal–hypophosphite frameworks tend to have unconventional tilts, columnar shifts, and off-center positions of the organic cations [33,34], which may induce ferroelectricity or other functional properties [33,34,35]. Furthermore, some exhibit magnetic order, although this feature strictly depends on the type of organic cation, e.g., [MHy]Mn(H_2_POO)_3_ has an antiferromagnetic order near 6.5 K, [FA]Mn(H_2_POO)_3_ shows an antiferromagnetic order below 2.4 K, and [EA] Mn(H_2_POO)_3_ (EA^+^ = ethylammonium) exhibits a magnetic order at 7.0 K [36,38].

**Figure 1:**
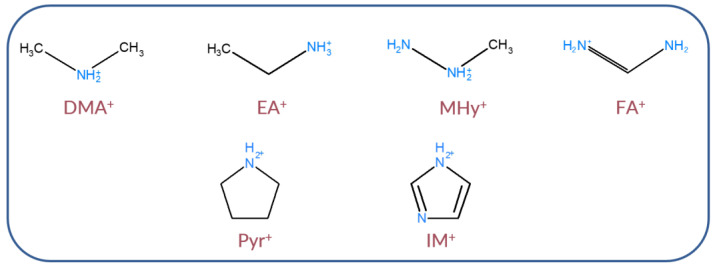
Chemical structures of organic cations used for the synthesis of [A]Mn(H_2_POO)_3_ hypophosphite perovskites.

Manganese and cadmium hypophosphites can also show bright and broadband photoluminescence (PL) [38]. The optical properties of divalent manganese strongly depend on the crystal field. Due to the 3d^5^ configuration, Mn^2+^ electrons are in the outer orbital and are sensitive to changes in their immediate surroundings. Mn^2+^ in the tetrahedral coordination exhibits green emission characteristics for the ion in a weak crystal field, whereas in the octahedral site (strong crystal field), it possesses red infrared emission. It is well-known that the type of A-site cation strongly influences the structural and optical properties of hypophosphite perovskites. For this reason, the photoluminescence (PL) properties of six manganese hypophosphites, namely [DMA]Mn(H_2_POO)_3_, [EA]Mn(H_2_POO)_3_, [MHy]Mn(H_2_POO)_3_, [FA]Mn(H_2_POO)_3_, [Pyr]Mn(H_2_POO)_3_, and [IM]Mn(H_2_POO)_3_ (DMA^+^ = dimethylammonium, Pyr^+^ = pyrrolidinium, and IM^+^ = imidazolium) were investigated. Four ammonium cations are aliphatic, one is cyclic, and one is aromatic. Figure 1 presents the structural formulae and ionic radii of chosen ammonium cations. This paper presents some general remarks on the influence of the organic cations on the *E_g_* value, PL band position, and thermal stability of the observed emission.

## 2. Results and Discussion

### 2.1. Crystal Structure

**Figure 2:**
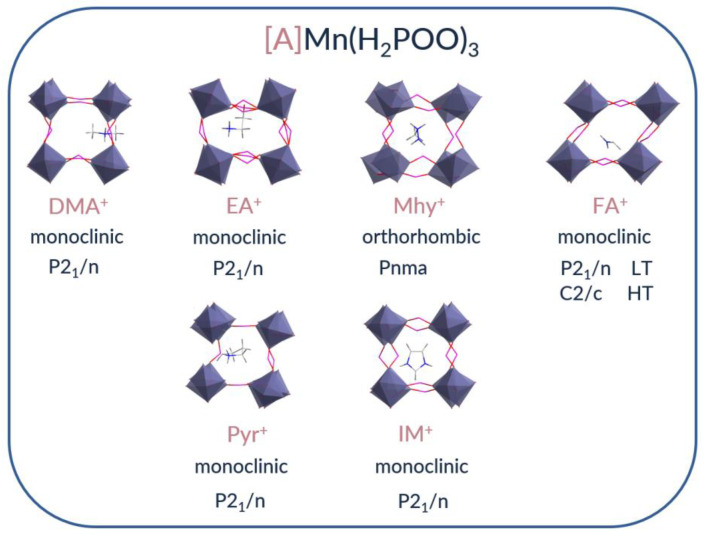
Crystal structures of the investigated manganese hypophosphites.

The difference in the ionic radius, the shape of the amine used, and the number of hydrogen bonds formed with the hypophosphite skeleton affect the crystal structure of the obtained compounds. Five of the six obtained compounds crystallize in the monoclinic system in the *P*2_1_/n space group. Among these compounds, [FA]Mn(H_2_POO)_3_ is unique because it transforms at 178 K into another monoclinic system with the *C*2/*c* space group (polymorph I). Manganese ions occupy three non-equivalent positions in the [DMA] Mn(H_2_POO)_3_ structure; two positions in the EA, IM, and Pyr analogs; and one position in the FA compound. [MHy] Mn(H_2_POO)_3_ is the only hypophosphite that adopts the orthorhombic *Pnma* symmetry, with the manganese ions occupying only one independent site. Figure 2 presents a visualization of the crystal structures of the investigated hypophosphites. Further details of structural properties can be found in recently published papers [35,37,38], and in the next paragraph, the influence of the distortion of the nearest Mn^2+^ environment and the number of occupied sites on the PL properties of manganese hypophosphites is discussed. The description of sample synthesis and measurement technique is shown in Supplementary Materials.

### 2.2. Spectroscopic Properties

**Figure 3:**
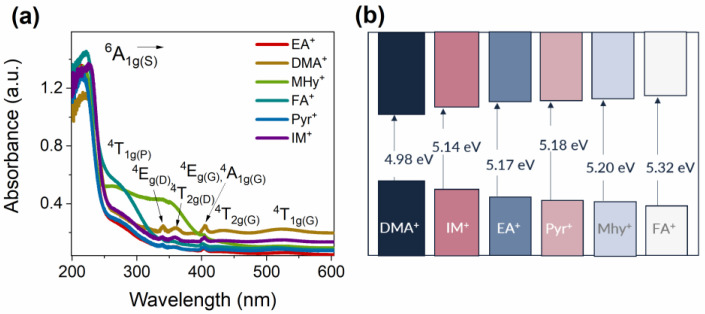
(**a**) Diffuse absorption spectra of the investigated samples and (**b**) scheme of the energy band gap estimated by the Kubelka–Munk function.

The absorption spectra of the investigated manganese hypophosphites were measured at room temperature. As can be seen in Figure 3a, they consist of strong host absorption at around 220 nm and less intense bands associated with the absorption of Mn^2+^ ions located in octahedral coordination. Thus, in Mn^2+^ ions, the absorption of the excitation energy occurs between the ^6^A_1g_ ground state and the ^4^T_1g_(P) (275 nm), ^4^E(D) (339 nm), ^4^T_2g_(D) (358 nm), ^4^A_1g_ and ^4^E_g_(G) (404 nm), ^4^T_2g_(G) (435 nm), and ^4^T_1g_(G) (524 nm) excited levels [15,17,39]. The position of these bands is clearly visible for all obtained manganese hypophosphite perovskites. The optical band gap (*E_g_*) was estimated using the Kubelka–Munk function. The determined *E_g_* values are presented in the scheme shown in Figure 3b and listed in Table 1. The smallest *E_g_* value of 4.98 eV is observed for [DMA]Mn(H_2_POO)_3_ and increases up to 5.32 eV for the [FA]Mn(H_2_POO)_3_ compound. At first glance, there is no relationship between the ionic radii of the ammonium cation and the *E_g_* value. However, a more careful analysis shows that some general conclusions can be drawn. First of all, the samples containing linear amines and the samples comprising pyrrolidinium and imidazolium should be analyzed separately. Furthermore, it appears that the ionic radii of the chosen organic cation are insignificant; however, the value of the Goldschmidt tolerance factor (*t_F_*) is of major importance. This geometrical parameter estimates the ion size mismatches that the perovskite structure is able to tolerate. To calculate the *t_F_* parameters for the investigated manganese hypophosphites, the modified Goldschmidt equation was applied as appropriate for HOIPs [31,32]:

(1) $$t_{F}=\frac{\left(r_{Aeff}+r_{xeff}\right)}{\sqrt{2}\left(r_{B}+0.5h_{xeff}\right)}$$

where *r_B_* denotes the ionic radii of cation *B* in the perovskite structure, and *r_ieff_* and *h_ieff_* are the effective ionic radii and the effective height of the used organic amine (*A*) and ligand (*X*), respectively. The determined *E_g_* values, together with the ionic radii of ammonium cations and *t_F_* values, are given in Table 1. As can be seen, the *E_g_* of [A]Mn(H_2_POO)_3_ increases as *t_F_* decreases. In other words, the optical band gap is smaller for a more perfect perovskite structure. However, this relation is not valid for the cyclic or aromatic amines, as [Pyr]Mn(H_2_POO)_3_ has a slightly higher value of *E_g_* than [IM]Mn(H_2_POO)_3_, in spite of its larger *t_F_*.

**Figure 4:**
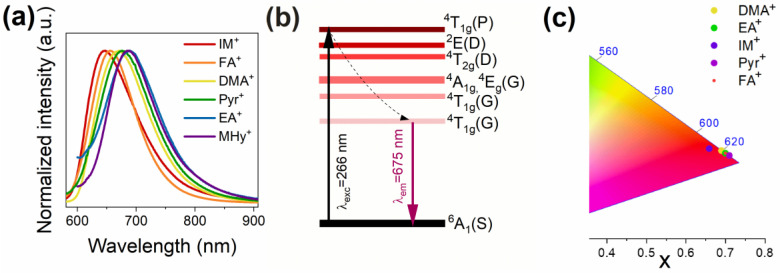
(**a**) Low-temperature PL spectra of [A]Mn(H_2_POO)_3_; (**b**) energy-level diagram of Mn^2+^ ions; (**c**) CIE coordinates of the investigated samples.

The normalized PL spectra of the prepared hybrid hypophosphites registered at 80 K are presented in Figure 4a. The PL bands are very broad and range from 600 nm to 900 nm. The full width at the half maximum (FWHM) value of these bands is approximately 2200 cm^−1^. After excitation under 266 nm, the electron moves to the ^4^T_1g_(P) excited level (Figure 4b). Then, as a result of non-radiative depopulation, the electron is transferred to the ^4^T_1g_(G) state. The radiative recombination of the electron from the ^4^T_1g_(G) level to the ^6^A_1g_(S) ground state generates intense red PL. The shape of the observed PL bands does not change with the applied organic cation, but the type of organic cation affects the band position (Table 1). The most redshifted PL is observed for the EA and MHy samples, with maxima at 688 nm and 686 nm, respectively. On the other hand, the emissions of the IM and FA compounds are positioned at 646 nm and 656 nm, respectively. PL of the DMA and Pyr analogs is located in the intermediate range, i.e., at 670 nm and 675 nm, respectively. It is worth mentioning that the emission of Mn-based dicyanamide perovskites was observed at lower wavelengths of about 626–629 nm, which is a result of using a shorter H_2_POO^−^ ligand instead of the long dicyanamide linkers [17,40]. The presented redshift of the emission is a natural consequence of the increasing crystal field strength acting on Mn^2+^ ions. The analysis of the manganese octahedron deviation (δ) from the ideal shape and position of the PL band shows a certain relationship between these parameters for the FA, EA, and MHy compounds (Table 1). In particular, if the Mn^2+^ ions are located in an almost perfect octahedron, and the strong crystal field causes a large splitting of energy levels and a significant redshift of the emission. [DMA]Mn(H_2_POO)_3_ deviates from this trend because the generated PL is a superposition of three emission bands belonging to Mn^2+^ ions located at three non-equivalent sites in the crystal structure. This trend is also observed for the IM and Pyr analogs. The considerable deviation (δ, 1.35%) of manganese octahedra in [IM]Mn(H_2_POO)_3_ shifts the emission band to a lower wavelength (λ_em_ = 646 nm), whereas almost perfect octahedra in [Pyr]Mn(H_2_POO)_3_ (δ is only 0.18%) lead to a redshifted emission at λ_em_ = 675 nm. Regardless of the observed changes in the position of the band maxima, all samples exhibit red emission at 80 K. The chromatic coordinates of the investigated compounds are demonstrated in Figure 4c.

**Table 1:** List of the most important parameters of [A]Mn(H_2_POO)_3_ perovskites, such as ionic radius (IR) of the ammonium cations [41], tolerance factor (*t_F_*), energy band gap (*E_g_*) [36,37,38], the position of the PL band at 80 K (*λ_max_*), octahedral deviation (δ) from the perfect shape [36,37,38], and quenching temperature (T_0.5_)_._

	DMA^+^	EA^+^	MHy^+^	FA^+^	IM^+^	Pyr^+^
IR (pm)	272	344	264	253	320	258
*t_F_*	0.90	0.905	0.891	0.858	0.869	0.90
*E_g_* (eV)	4.98	5.17	5.20	5.32	5.14	5.18
*λ_max_* (nm)	670	688	686	656	646	675
δ (%)	0.17	0.39	0.44	10.6	1.35	0.18
T_0.5_ (K)	100	130	122	135	135	115

**Figure 5:**
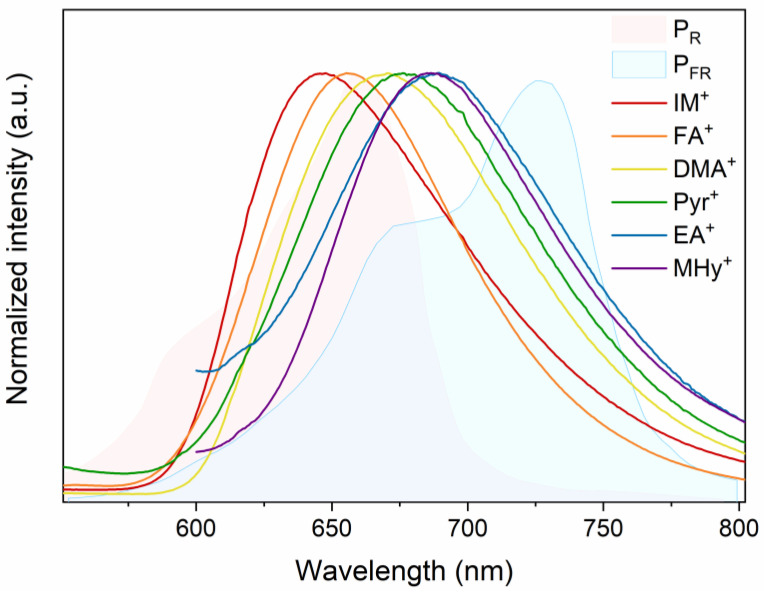
Collation of PL spectra of the investigated manganese hypophosphite and absorption of the P_R_ and P_FR_ phytochromes.

**Figure 6:**
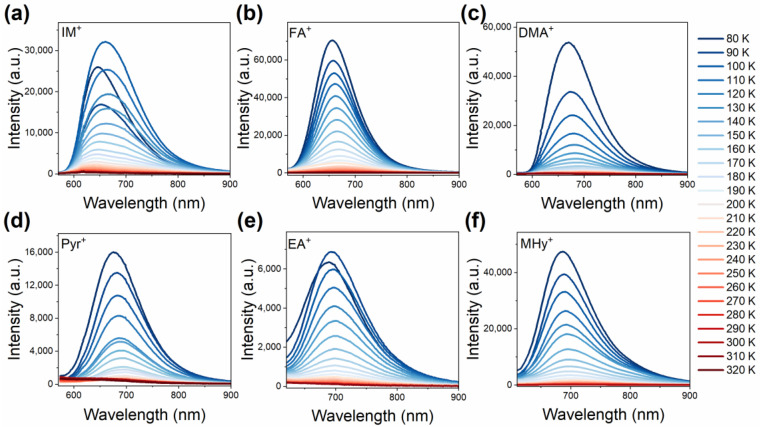
(**a**–**f**) Temperature-dependent emission spectra of the investigated manganese hypophosphite perovskites.

**Figure 7:**
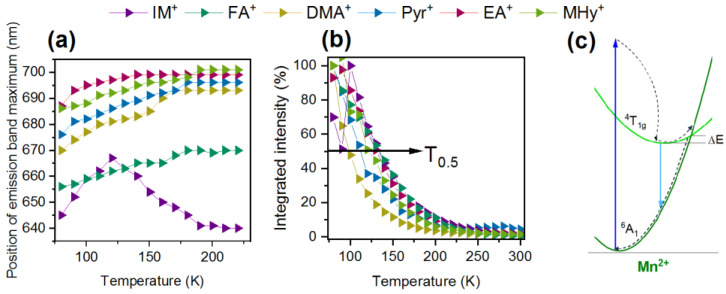
(**a**) Position of the PL band as a function of temperature up to 220 K; (**b**) integrated emission intensity of [A]Mn(H_2_POO)_3_ as a function of temperature; and (**c**) energy-level diagram of Mn^2+^ presenting the mechanism of PL quenching.

The broad emission localized in the near-infrared region can be attractive for plant growth. The absorbed photoenergy can be used by plants in the photosynthesis process. The main photopigments found in plants are chlorophyll A, chlorophyll B, phytochrome P_R_, and phytochrome P_FR_ [42]. The first two types of photopigments mainly absorb blue light (400–500 nm), whereas P_R_ and P_FR_ are sensitive to red and far-infrared light, respectively [42]. As can be clearly seen in Figure 5, the emission generated from Mn-based hybrid hypophosphite perovskite covers almost the entire absorption range of the P_R_ and P_FR_ phytochromes. Nevertheless, it appears that [DMA]Mn(H_2_POO)_3_ emission coincides to the greatest extent with the absorption of both types of phytochrome. The biggest disadvantage is the low thermal stability of [A]Mn(H_2_POO)_3_ sample emissions (Figure 6 and Figure 7). At the moment, it seems impossible to construct a device that would keep the hypophosphite crystal at the temperature of 80 K.

The temperature-dependent PL spectra of the investigated samples recorded from 80 K to 320 K every 10 K are presented in Figure 6. Almost all hypophosphites exhibit the highest emission intensity at 80 K. However, the thermal stability of [IM]Mn(H_2_POO)_3_ and [EA]Mn(H_2_POO)_3_ emissions is quite different. At the beginning, the intensity of Mn^2+^ emission decreases and then increases. This abnormal thermal behavior can be explained by energy transfer between the Mn^2+^ ions located at two independent optical sites [36]. A common feature of all investigated perovskites is the asymmetric shape of the PL band, moving the band position to a higher wavelength with sample heating (Tab 7a). This behavior can be associated with the Mn^2+^ ions occupying more than one optical site and small changes in the crystal field strength with increasing temperature [23,36,43,44]. A careful analysis of the obtained results shows that the emission of the [DMA]Mn(H_2_POO)_3_ analog quenches much faster than the remaining samples. The quenching temperature, defined as the temperature at which intensity drops to half the initial value (T_0.5_), is 100 K for the DMA compound (Figure 7b). [Pyr]Mn(H_2_POO)_3_ and [MHy]Mn(H_2_POO)_3_ exhibit significantly better thermal stability, with T_0.5_ values of 115 K and 122 K, respectively. However, the emission of Mn^2+^ ions in the samples comprising EA^+^, FA^+^, and IM^+^ has the highest thermal stability. The emission of manganese ions is quenched by the crossing of the ^4^T_1g_(G) excited state with the parabola of the ^6^A_1_(S) ground level (Figure 7c). Nevertheless, temperature-dependent measurements proved that Mn^2+^ ion emission in the hypophosphite perovskites quenched faster than in the case of the Mn-based dicyanamides.

**Figure 8:**
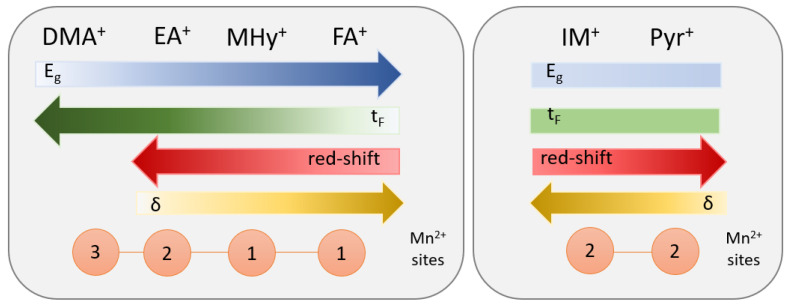
Influence of the amine applied in [A]Mn(H_2_POO)_3_ compounds on structural and optical features. The scheme shows the correlation between the structural parameters (such as *t_F_*, octahedral deviation (δ) from the perfect cubic shape, and the number of Mn^2+^ sites) and the optical properties (energy band gap and PL position).

The presented results explain the influence of some key structural factors on the optical properties of the family of Mn-based hybrid hypophosphites. Figure 8 summarizes the correlation between structural parameters, such as *t_F_*, octahedral deviation (δ), the number of Mn^2+^ sites, and optical features. As can be seen, the compounds comprising linear cations should be analyzed separately from those with cyclic organic cations. Furthermore, there is no doubt that there is a correlation between the Goldschmidt tolerance factor and the energy band gap value. The higher the *t_F_* value, the lower the *E_g_*, so the optical band gap decreases for the samples with an almost ideal perovskite structure. For the IM and Pyr analogs, the *t_F_* and *E_g_* values are comparable; therefore, it is impossible to define any rules. Analysis of the results allowed for the correlation of the octahedral deviation with the position of the PL band. The shift of the PL band is greater for manganese ions occupying the almost perfect octahedron. The results reported for [DMA]Mn(H_2_POO)_3_ do not comply with this rule due to the three independent optical sites of Mn^2+^ ions.

## 3. Conclusions

This paper presents the results of an investigation on the effect of organic cations on the PL properties of Mn^2+^ ions. Mn-based hybrid hypophosphites were synthesized by crystallization from solution. Five of the six obtained compounds crystallize in the monoclinic system in the *P*2_1_/*n* space group. The only exception is [MHy]Mn(H_2_POO)_3_, which adopts an orthorhombic structure (*Pnma* space group). The obtained results indicate that the type of ammonium cation has a significant impact on the Goldschmidt tolerance factor value and the octahedral deformation parameter. As a consequence, the size of the energy band gap and the position of the emission band are tuned. It was found that the samples with a higher tolerance factor have a smaller energy band gap. Moreover, the reported redshift of the PL band position of the investigated compounds is associated with a smaller octahedral deformation. However, the temperature-dependent measurements prove that Mn^2+^ emission in the hypophosphite perovskites quenches faster than the PL of the Mn-based dicyanamides. The broad red PL of Mn^2+^ ions assigned to the ^4^T_1g_(G) → ^6^A_1g_(S) transition overlaps with the absorption of the phytochrome P_R_ and the phytochrome P_FR_. Therefore, the investigated hypophosphites may be applied in an indoor lighting system for plant growth. However, a considerably disadvantage of these compounds is the low thermal stability of their PL.

## Data Availability

Not applicable.

## Supplementary Materials

The following supporting information can be downloaded at: www.mdpi.com/article/10.3390/molecules27248953/s1. Description of sample synthesis and measurement technique.

## Sample Availability

Samples of the compounds are available from the author.
