# Supplementary material for: Effect of Organic Cation on Optical Properties of [A]Mn(H2POO)3 Hybrid Perovskites
Source: Molecules. 2022 Dec 15;27(24):8953. doi: 10.3390/molecules27248953 (PMC9784195; doi:10.3390/molecules27248953)
Supplement: Supplementary file 1 [file molecules-27-08953-s001.zip › molecules-2065237-supplementary.pdf]

Supplementary Information for

Effect of organic cation on optical properties of $[A]Mn(H_2POO)_3$ hybrid perovskites

Dagmara Stefańska

W. Trzebiatowski Institute of Low Temperature and Structure Research of the Polish Academy of Sciences, Wroclaw, Poland;

* Correspondence: D.Stefanska@intibs.pl

Samples synthesis

Single crystals of $[DMA]Mn(H_2POO)_3$, $[EA]Mn(H_2POO)_3$, $[MHy]Mn(H_2POO)_3$, $[FA]Mn(H_2POO)_3$, $[Pyr]Mn(H_2POO)_3$, and $[IM]Mn(H_2POO)_3$ were obtained by the crystallization from the solution. The solution of metal carbonate (5 mmol) in 12.92 ml (120 mmol) of hypophosphorous acid was stirred and kept at 50°C for a few minutes. Then 15 mmol of the appropriate amine was added and the mixture was left at 50°C in the air. The faint pink crystals were grown overnight and were separated from the mother liquid, washed with the methanol, and dried at room temperature.

Measurement Techniques

The XRD spectra were measured with an X'Pert PRO powder diffractometer (PANalytical, The Netherlands) equipped with a linear PIXcel detector and using Cu Kα radiation (λ=1.54056 Å). The RT absorption spectra of the powdered samples were measured using a Varian Cary 5E UV–vis–NIR spectrophotometer. The emission spectra at various temperatures under 266 nm excitation from a diode laser were measured with the Hamamatsu photonic multichannel analyzer PMA-12 equipped with a BT-CCD linear image sensor. The temperature of the single-crystal was controlled using a Linkam THMS 600 Heating/Freezing Stage.
